# Psychosocial Care Needs of Melanoma Survivors: Are They Being Met?

**DOI:** 10.1371/journal.pone.0132754

**Published:** 2015-08-21

**Authors:** Sabine Fischbeck, Barbara H. Imruck, Maria Blettner, Veronika Weyer, Harald Binder, Sylke R. Zeissig, Katharina Emrich, Peter Friedrich-Mai, Manfred E. Beutel

**Affiliations:** 1 Department of Psychosomatic Medicine and Psychotherapy, Medical Psychology and Medical Sociology, University Medical Center of the Johannes Gutenberg-University Mainz, Mainz, Germany; 2 Department of Psychosomatic Medicine and Psychotherapy, University Medical Center of the Johannes Gutenberg-University Mainz, Mainz, Germany; 3 Institute for Medical Biometrics, Epidemiology and Informatics (IMBEI), University Medical Center of the Johannes Gutenberg-University Mainz, Mainz, Germany; 4 Institute for Medical Biometrics, Epidemiology and Informatics (IMBEI), Cancer Registry, University Medical Center of the Johannes Gutenberg-University Mainz, Mainz, Germany; Supportive care, Early DIagnosis and Advanced disease (SEDA) research group, UNITED KINGDOM

## Abstract

Patients who have survived malignant melanoma for more than five years may lack the opportunity to talk about their burden. As a consequence their psychosocial care needs remain undetected and available supportive interventions may not be utilised. Therefore, the psychosocial burden of this patient group needs to be assessed using specific screening instruments. The aim of this study was to investigate the psychosocial burden of long-term melanoma survivors, their psychosocial care needs and the determinants of these needs. We wanted to find out if the use of professional support corresponds to the care needs defined by experts. Using the cancer registry of Rhineland-Palatinate, melanoma patients diagnosed at least 5 years before the survey were contacted by physicians. N = 689 former patients completed the Hornheide Questionnaire (short form HQ-S) to identify psychosocial support need (scale cut off ≥ 16 or item-based cut-off score) and the potential psychosocial determinants of these needs. Additionally, they were asked about their utilisation of the professional support system. More than one third (36%) of them was in need for professional psychosocial support. The highest burden scores concerned worry about tumour progression. Younger age (< 50), higher general fatigue, higher symptom burden, lower general health, negative social interactions and unfulfilled information needs were significant predictors of the need for psychosocial intervention. Related to the percentage of survivors identified as ‘in need’, the professional support system was underused. Further studies should investigate whether using the HQ-S to routinely identify burdened melanoma patients could lead to better fulfilment of their intervention needs, ultimately enhancing health-related quality of life.

## Introduction

According to latest information from the German Cancer Registration in 2010 19.220 people were diagnosed with malignant melanoma in Germany, 9640 women and 9580 men. Therefore, malignant melanoma accounted for 4.3% and 3.8% of malignant tumours in women and men, respectively [1]. Since the 1980s the incidence rate has tripled. This can be explained by changes in leisure time behaviour of light-skinned people and increasing solar exposition of the skin [2]. In view of this malignant melanoma shows the most precipitous increase of a preventative carcinoma and the most frequent cancer in middle-aged adults [2].

The success of treatment is highly dependent on the tumour staging. Approximately 90% of malignant melanomas are diagnosed as a primary tumour without detectable metastases (stage I or II). Therefore, the 10-year survival rate of 75% to 80% is comparatively good. The median survival of patients with a metastatic carcinoma (stage IV), however, is only 6 to 7 months [3]. Depending on tumour localisation, surgical intervention can cause severe disfigurements, especially in the head and neck area [4]. Tumour progression remains an on-going threat for the survivors. Within 10 years, this occurs in approximately 6–14% of cases, including cases of early stage carcinomas and after successful primary treatment [4].

Patients treated for melanoma should receive life-long aftercare. Clinical guidelines recommend regular follow-ups, consisting of a full body skin examination by a physician, complemented by regular patient or partner-assisted self-examination [5]. However, internationally there is poor consensus regarding medical and psychosocial follow-up care [6]. The guidelines of the German Society of Dermatology and the German Cancer Societies recommend an aftercare regimen of more than 10 years, including psycho-oncological care. Whereas the medical aftercare is specified, there are no clear recommendations for psychosocial screening or interventions, with the exception of the recommendation that patients at risk should be referred for professional psychosocial support [7]. Little is known about if and how medical and psychosocial follow-up care are used by long-term survivors of malignant melanoma. A systematic literature review [8] found only 31 empirical psycho-oncological studies of malignant melanoma since 1990, and most were methodically weak. Data on psychosocial care needs were very inconsistent.

In a systematic review of published studies, Kasparian et al. [9] found that approximately 30% of all patients diagnosed with melanoma, including long-term survivors, reported levels of psychological distress indicative of the need for clinical intervention. Based on the Hornheide Questionnaire, a need for psychosocial intervention has been identified in 45% melanoma patients out of a sample (n = 846) of patients with skin and head tumours. This prevalence was higher than in samples of squamous cell carcinoma (39%) and basalioma (34%) patients. Fear of recurrence, lack of professional support, distress, nervousness, job-related stress, reduced self-confidence and subjective well-being were the main reasons for being in need of intervention [10]. As Winzer et al. [11] pointed out, cancer patients´ perceived (self-assessed) need for psychosocial support may be different from the assessment of the health care professionals. Also, an individual who expresses a need for support may not necessarily access help. They did not find any relation between subjective and normative need, but the data partly rely on a very small sample; only 9% of melanoma patients identified a need for psychosocial intervention.

According to Schulz et al. [12] patient use of intervention options can be conceptualised as a complex multistep decision procedure, from the perception of a problem to the individual decision to access professional help. Based on different cancer entities, a number of studies have identified social and clinical variables as predictors of psychosocial intervention needs. The need was more pronounced when the illness was advanced or metastatic [13, 14, 15, 16], e. g. tumour size greater than 4 mm, affecting the head and neck area and diagnosed less than 3 years ago [13]. Cancer patients were more likely to need support when they were female [13, 17, 18], of younger age [13, 17], belonged to a higher social class [17], were without a partner [13] and were less educated [14]. Insufficient physician support [15], low social support [15] and detrimental interactions with others [14] were also determinants of care needs. High symptom burden, low quality of life [14], fatigue [17], an avoidant [17] or depressive coping style [19, 20] were additionally associated with a need for care. The same was true for fear of progression [15, 19, 20], higher psychosocial burden, comorbid depression or anxiety and the feeling of being insufficiently informed about the illness and supportive institutions [19, 20].

Self-assessments of melanoma patients regarding their burden differed from the assessments of their physicians [21]. Care needs can be underestimated and remain unmet, so a systematically psychometric screening is necessary to prevent this. Indeed, many melanoma patients desired additional consultations with a physician (59%) or a psychotherapist (20%), indicating that their needs for care had not been recognized [19]. As we could show recently, distress ratings of melanoma patients (a total of 47%; [22]) by far exceeded distress identified by physicians (in 7%; [23]); thus, it can be surmised that care needs are underestimated and remain unmet. Thus, a systematical psychometric screening may be required in order to detect patients’ need for care.

The purpose of MeLa, across-sectional registry-based study, was to determine the prevalence of burden and need for psychosocial support of melanoma survivors. The study examined predictors of psychosocial intervention needs, taking into account illness characteristics, demographic data, health-related quality of life, fatigue, coping with illness, social support, comorbidity of depression and anxiety disorder and information needs. We also investigated whether the need for psychosocial intervention based on a standardised scale is associated with the utilisation of the psychosocial health care system. We assumed that psychosocial care was underused compared with needs identified. We further that patients with high psychosocial burdens use the professional support system more often than the patients under the cut-off score.

## Patients and Methods

### Study design and participants

All patients with a diagnosis of malignant melanoma (ICD-10: C43) who had been registered by their dermatologist (n = 112) with the Cancer Registry of Rhineland-Palatinate as part of routine documentation were eligible. Inclusion criteria were a) registration in the time period from 2000 to 2005, b) age of 14 years or older at diagnosis and signed written informed consent for study participation. We excluded those patients a) who were not informed about their registration and b) did not have adequate German language comprehension or cognitive ability to participate. For legal reasons and in order to insure confidentiality, only the Cancer Registry was allowed to decode the name of the patient. Only the dermatologist who had originally registered the patient was eligible to contact the patient, who was then provided with a written informed consent form and the questionnaire. Patients who did not react to the letter within six weeks received a reminder letter and were contacted once more. Those who still did not respond were not contacted further. Data analysis was performed with coded data without reference to any personal identification.

Two thirds (67%) of the dermatologists took part. They requested questionnaires for n = 1702 patients, representing 80.5% of all eligible patients (n = 2113). Of all contacted patients (n = 1320), n = 689 questionnaires were returned. Thus, 52.2% of the contacted patients took part, representing 32.6% of registered patients. We could not reach 19.4% of the patients because the corresponding dermatologists were not reachable.

### Ethics statement

The protocol was approved by the Ethics Committee of the Statutory Physician Board of the State of Rhineland Palatinate (Reference number 837.161.11, 7703).

### Measures

Sociodemographic data were obtained using a standardised self-reporting questionnaire. Illness-related information (UICC stage, tumour site, time since diagnosis) was extracted from the Rhineland-Palatinate Cancer Registry.

Psychosocial care needs were assessed by using the 9 Item Short Form of the Hornheide Questionnaire (HQ-S) [24], including physical and mental wellbeing, tumour-related anxiety, tension, self-esteem, social support, physician support and the information needs of skin cancer patients. This reliable measure (Cronbach´s α = .87 in the MeLa data set) has been developed for skin cancer patients and has shown good validity [25, 26]. The questionnaire allows for the identification and classification of patients in need of professional psychosocial or psycho-oncologic intervention. The degree of the distress is measured on a scale from 0 "does not apply" to 5 = "applies and troubles me extremely”. Item cut-off scores for each distress dimension and a cut-off score for the scale sum (≥ 16) were developed by the author of the test and validated by clinical interviews in order to classify the need for psychosocial intervention.

Depression was measured using the German version of the Patient Health Questionnaire Depression Module (PHQ-9 [27]; Cronbach´s α = .86 in the MeLa data set). Categorisation of depression is defined as a score of PHQ-9 ≥ 10, indicating moderate to severe depressive symptoms for the past two weeks [28]. Sensitivity is 92% for major depression (cut-off ≥ 10) in primary care, while specificity is somewhat lower (80%) [29].

The Generalized Anxiety Disorder Questionnaire (GAD-7) of the PHQ was used to identify probable cases of generalised anxiety disorder and to assess symptom severity. The GAD-7 is based on the most prominent diagnostic features of the DSM-IV diagnostic criteria for generalised anxiety disorder. Seven items are scored on a four-point Likert scale over the past two weeks from 0 (“not at all”) to 3 (“nearly every day”), with a total score ranging from 0 to 21. Reliability, validity and standard values were established using a representative German population sample (n = 5036; age 48 ± 18 years, 54% female [30]. Internal consistency in the MeLa data set is Cronbach´s α = .89. The EORTC Quality of Life Core Questionnaire EORTC-QLQ-C30 [31] measures cancer-related quality of life reliably and with good validity using five functional scales (physical, role, cognitive, emotional, social; in the MeLa data, Cronbach´s α = .80/.90/.68/.89/.85 in order of the previous list), three symptom scales (pain, fatigue, nausea/vomiting; in the MeLa data set Cronbach´s α = .89/.85/.33), a global health and quality of life scale, and six single items assessing additional symptoms commonly reported by patients (e.g. appetite loss, sleep disturbance) as well as the perceived financial impact of the disease and treatment. The EORTC QLQ-C30 consists of 30 items that are scored on 4-point Likert scales, ranging from 1 (“not at all”) to 4 (“very much”). Two items in the global health and quality-of-life subscale are scored on a 7-point linear analogue scale. All functional scales and individual item scores are transformed to a 0–100 scale.

Fatigue was assessed using the Multidimensional Fatigue Inventory (MFI), an internationally validated, multidimensional self-administered instrument [32]. Its 20 items form the following subscales (Cronbach α in the MeLa data set in parentheses): General (α = 81) physical (α = .86), and mental fatigue (α = .81), reduced motivation (α = .65), and reduced activity (α = .84). Each subscale contains four items (range from 4 to 20, with higher scores indicating increased fatigue). The German version shows moderate to good reliability Convergent validity was moderate for aspects of quality of life [33].

The 24-item German version of the Illness-specific Social Support Scale (ISSS) measures emotional, informational and practical support [34]. The two scales”positive social support” (15 items) and “detrimental interaction” (9 items) are scored on a 5-point Likert scale ranging from 0 (“never”) to 4 (“always”). The authors report a high reliability and good factorial validity. Internal consistency in the MeLa data set is Cronbach´s α = .94 for the positive social support scale and .72 for the detrimental interactions scale. Construct validity is supported by positive correlations to selected variables of social interaction.

The Brief Cope (BC) is a short version of the COPE questionnaire [35, 36], which has proven useful in health-related research. It consists of 14 coping scales with a Likert scale (1 = “never” until 4 = “very often”), each with two items (e. g. “self-distraction”, “active coping”, “denial”, “substance use” and “use of emotional support”). Due to the fact that more than half of the scales have a Cronbach Alpha coefficient under .70, we decided to perform a cross validation in our sample. An exploratory factor analysis with the Brief Cope items led to three interpretable coping scales with acceptable reliability (accounting for 38% of total variance). The first dimension could be labelled as “seeking external support” (α = .75), the second “denial/self-blaming” (α = .74), and the third “active coping” (α = .76). The dimensions are in good accordance with other Brief-Cope-related analytic results [37, 38].

### Service and information needs

Since there were no standardised scales for measuring service and information needs for melanoma patients, we developed nine dichotomised single questions (“yes/no”) addressing main domains [14, 19] of support sources (psychological counselling, social counselling, pastoral care, self-help group, cancer care counselling, telephone counselling, internet counselling, internet forum with concerned persons). For measuring information needs we did not find a suitable standardised questionnaire and developed an ad hoc scale with 15 illness, therapy or psychosocial care related items covering main aspects: diagnosis, chance of recovery, course of the disease, treatment, side effects, other therapy options, course of the therapy, follow-up care, genetic risk, prevention, rehabilitation, psychosocial support, self-help groups, participation in studies, and a second opinion. Patients rated on Likert scales with five steps (1 = “not at all” to 5 = “very much”) about how adequately they felt informed about the issues. Ratings were dichotomised (0 = rather/much informed: no information deficit; 1 = not at all/little/a bit informed: information deficit) to determine the percentage of patients with an information deficit.

### Statistical analysis

For descriptive analysis, absolute and relative frequencies were computed for the categorical variables. Missing data were replaced according to the recommendations of the test authors. Associations between the standardised questionnaires were determined using Spearman´s correlation coefficients in order to determine the validity of the HQ-S. For the categorisation of ‘being in need’ of psychosocial intervention, the HQ-S item cut-off score and the sum score cut-off (calculated from the item scores) were used. Social class was determined using a common index [39]. In order to estimate the effects of the socio-demographic data (gender, age, marital status, education, social class, urban/rural destination), social variables (partnership/the medical issues (UICC status, time since diagnosis), comorbidity (PHQ-9, GAD-7) and psychosocial variables (scales of the MFI, Brief COPE, ISSS; EORTC-Symptoms, EORTC-Global Health) on the care needs (HQ-S), a logistic regression model was fitted. We present adjusted odds ratios, the corresponding 95% confidence intervals and p-values. Because of missing values in the potential predictors, a multiple imputation was performed [40]. We used a multiple imputation with k = 10 imputed data sets via the SAS procedure MI. For two randomly chosen single imputation data sets, we selected a set of covariates with a logistic regression model using forward and backward selection with a selection level of 5%.

For each single imputation data set, we then performed logistic regression models for the previously selected covariates and combined the results via the SAS procedure MIANALYZE. This logistic regression model was used for confirmatory analyses.

Statistical analysis was performed with the Statistical Package for the Social Sciences (SPSS, Chicago, IL, USA, version 21.0) and SAS 9.2 for Windows 9.2 TS Level 1M0 (SAS Institute Inc. Cary, NC, USA).

## Results

### Sample

Our sample included 689 malignant melanoma survivors (354 women and 335 men; see Table 1). Nearly two thirds of the participants were 50 years old or older. Most were diagnosed at stage UICC 1 (no spread or distant sites, 53%) and the melanoma was located in the extremities (47.7%); 97.1% had melanoma surgery. At the time of the study, 69.5% had lived with their illness for 6 to 9 years, 31.5% 10 years or more. Most were married (75.5%), 82.7% had a partner and lived in rural communities (72%). Regarding education, 48.3% had attended primary, 28.4% secondary and 20.6% senior high school. Social class was equally distributed among lower (34, 4%), middle (34%) and upper social class (31.6%)

**Table 1:** Demographic and medical characteristics of the sample (n = 689).

	n	%
**Age**		
< 39	52	7.6
40–49	111	16.1
50–59	137	19.9
60–69	133	19.3
70–79	184	26.7
≥ 80	72	10.5
**Gender:** male	335	48.6
**Marital status**		
Single	49	7.1
Married	520	75.5
Separated/divorced	49	7.1
Widowed	70	10.2
**Partnership:** yes	570	82.7
**Educational level**		
Primary school	333	48.3
Secondary school	196	28.4
High School	142	20.6
Other	17	2.5
**Residence:** rural	496	72.0
**Time since diagnosis (years)**		
6	104	15.1
7	139	20.2
8	129	18.7
9	107	15.5
10	96	13.8
11	88	12.9
12	26	3.8
**Social class**		
Lower	237	34.4
Middle	234	34.0
Higher	218	31.6
**UICC stage at diagnosis** ^1^		
1	365	53.0
2	34	4.9
3	7	1.0
Unknown	283	41.1
**Melanoma surgery:** yes	660	97.1
**Tumour site ICD-10 (malignant melanoma)**		
extremities (C43.6, C43.7)	329	47.7
trunk (C43.5)	248	36.0
head and neck (C43.2-C43.4)	93	12.5
overlapping skin/not otherwise specified (C43.8, C43.9)	19	2.8

^1^UICC-stage: till 2003 (year of diagnosis) according to TNM 5. ed., Springer publisher 1997, from 2004 (year of diagnosis) TNM 6. ed., Springer publisher 2002; classification according to TNM 6 in A and B were subsumed to the particular stage; missing data: marital status (1), partnership (23), educational level (1), melanoma surgery (9)

### Need for psychosocial support

Based on the HQ-S scale sum cut-off score (≥ 16), 14% of the sample were identified as in need of psychosocial intervention. When additionally cut-off scores were considered for each individual item, the care need rose to 36%. As can be seen in Table 2 melanoma survivors were primarily burdened by fear of recurrence (M = 1.4) or being anxious (M = 1.2). Fear that people will reject them because of their altered appearance (M = 0.2) or not having confidence in their ability to resume or continue their normal work (M = 0.4) were the lowest burdens.

**Table 2:** Item and scale analysis of the Hornheide Questionnaire short form (HQ-S).

Items	%^1^ ^,^ ^3^						M	SD	r_it_	n
	0	1	2	3	4	5				
1. I am often anxious.	42	27	14	10	4	3	1.2	1.35	.73	683
2. I cannot relax and rest.	57	18	9	8	6	2	0.9	1.36	.69	680
3. I am afraid of life with the disease.^2^	58	22	9	5	3	3	0.8	1.24	.72	677
4. I do not have confidence in my ability to resume or continue my normal work.	81	9	3	3	1	3	0.4	1.08	.52	672
5. I feel physically less productive than prior to falling sick.	71	13	5	4	4	3	0.7	1.29	.63	679
6. The thought of the tumour recurring makes me afraid.^2^	40	28	9	9	6	8	1.4	1.58	.67	683
7. I fear that people will reject me because of my altered appearance.	88	9	1	1	0	1	0.2	0.73	.44	680
8. Talking to my close relatives about my sorrows and fears is difficult.	68	16	6	4	3	3	0.7	1.25	.56	680
9. I feel insufficiently informed about my disease and the treatment.	75	11	5	5	3	1	0.6	1.14	.51	680

^1^6-point Likert Scale from 0 = “does not apply" to 5 = “applies and troubles me extremely”, Cronbach´s α = .87”;r_it_ = discrimination power; item cut-off scores (care need prevalent) underlined

^2^a care need prevalent, if the sum of the anxiety measuring items (3, 6) is 7, 8, 9 or 10

^3^Care need index: sum score ≥ 16: 14% of the sample, M = 6.78, SD = 7.85; need for psychosocial intervention (HQ-S ≥ 16 or underlined item cut-off scores), prevalent 36% (n = 239), not prevalent 64% (n = 433)

### Predictors of psychosocial care needs

We hypothesised that the need for psychosocial care (based on HQ-S scale cut off ≥ 16 or item cut-off scores) is determined by age, level of school education, fatigue, illness-related social support and time since initial melanoma diagnosis. The results of the multivariate logistic regression are shown in Table 3. “Compared to the youngest group, the need for psychosocial care strongly declined with age”Higher general fatigue (MFI-general), higher symptom burden (EORTC QoL-Symptoms), lower general health (EORTC QoL-General Health), detrimental social interactions (ISSS), a denial/self-blaming coping style (Brief COPE) and high information needs were significant predictors of being in need for psychosocial intervention. Gender, partnership, anxiety (GAD-7), depression (PHQ-9), fatigue (MFI), a support seeking or active coping style (Brief COPE), positive social support (ISSS), disease stage, time since diagnosis, social class and living in a rural or urban were not significant.

**Table 3:** Predictors of the need for psychosocial care: Multivariable logistic regression based on multiple imputation (10 imputation data sets, Nagelkerkes R^2^ .41-.44).

	*Odds ratio*	95% Confidence Limits		P
Age group < 50				
Age 50–69	0.54	0.32	0.90	0.0180
Age ≥70	0.32	0.18	0.55	< .0001
General Fatigue (MFI)	1.12	1.04	1.20	0.0015
Quality of life—Symptoms (EORTC-QoL-C30)	1.02	1.00	1.05	0.0259
Quality of life—General Health (EORTC-QoL-C30)	0.98	0.97	1.00	0.0087
Detrimental Interactions (ISSS)	1.55	1.05	2.29	0.0258
Denial/Self-blaming (BC)	4.57	2.38	8.76	< .0001
Lack of information	1.06	1.02	1.10	0.0041

* Multiple Imputation for a set of covariates selected by forward and backward selection (level of selection 5%) in single imputation data

### Health care utilisation

As can be seen in Table 4, many of the long-term survivors (22%) reported no medical aftercare consultation, although follow-up visits should be carried out on a yearly basis with this group of patients. Most of them have not used the psychosocial care system or lacked the possibilities to do so: the rate of utilisation does not exceed 7% (n = 47 psychological counselling, n = 46 psychopharmacologic drugs), and even fewer have used psychotherapy (4%, n = 25). Only 13% (n = 46) used one or more kind of professional psychosocial/psycho-oncological help. Survivors in need of professional psychosocial intervention (HQ-S) showed higher probabilities for using the psychosocial support system (odds ratios 5.19–10.30, lowest for any psychosocial support), but a lower probability for getting medical aftercare (odds ratio 0.74).

**Table 4:** Need for psychosocial care (HQ-S) and use of the professional support system (medical, psychosocial), n = 644–659.

Using Support		HQ-S above cut off				*χ* ^,^, *p*	*Odds Ratio*	Confidence Limits	
		Yes 236 (36%)	No 423 (64%)						
Dermatological aftercare	yes	175	335	510	78%				
	no	59	84	143	22%		0.74	0.51	1.09
	sum	234	419	653		2.34, ≤ .05			
Psychological counseling	yes	39	8	47	7%				
	no	196	414	610	93%		10.30	4.72	22.45
	sum	235	422	657*		49.11, ≤ .001			
Psychotherapy (weekly)	yes	20	5	25	4%				
	no	217	417	634	96%		7.69	2.85	20.76
	sum	237	422	659*		21.88, ≤ .001			
Any psychosocial care**:	yes	59	26	85	13%				
	no	170	389	559	87%		5.19	3.16	8.52
	sum	229	415	644*		48.97, ≤ .001			
Psychopharmacologic drugs	yes	34	12	46	7%				
	no	202	411	613	93%		5.76	2.92	11.37
	sum	236	423	659*		31.23, ≤ .001			

2 x 2 contingency tables, Chi-square tests, odds ratios

*gap to n = 689 due to missing data,

**use of one or more of the following types: psychological counseling, social counseling, pastoral care, self-help group, cancer care counseling, telephone counseling, internet counseling, internet forum (concerned persons)

## Conclusions

Given the paucity of data, this study was conducted to (1) identify the psychosocial care needs of long-term survivors of malignant melanoma, (2) the determinants of these psychosocial care needs and then (3) compare expert-defined needs for psychosocial intervention with self-reported utilisation of the health care system.

### Prevalence of need for care

The need for psychosocial intervention was based on a standardised measure validated for malignant melanoma, the short form of the Hornheide Questionnaire. After applying the expert-defined threshold, which Strittmatter [24] determined in his standardisation group, we found that 36% of our sample was in need of psychosocial support. This is in concordance with the prevalence data Kasparian et al. [9] found in an international metaanalysis based on studies with recently diagnosed melanoma patients. The prevalence was lower than Strittmatter et al. [41] found with the HQ (45%) in a melanoma sample and much higher than Winzer et al. [11] detected in a small sample, where 9% of outpatients needing professional psychosocial care were found with this instrument. It cannot be precluded that the low cut-off of the HQ-S, which categorises patients as in need when only one item is rated above a certain cut-off, inflates the need prevalence. However, this criterion might be very sensible for the detection of the patient´s burden. Our results indicate that the psychosocial burden (and the care needs) of melanoma patients persists for many years after diagnosis.

### Determinants of care needs

As the multivariate approach shows, compared to those without a need, patients who were identified as in need for psychosocial support are characterised by younger age (< 50), higher general fatigue (MFI), higher symptom burden (EORTC), lower general health (EORTC), suffering from detrimental interactions (ISSS), a denial/self-blaming coping style (Brief COPE) and a lack of illness related information; the coping style variable had the highest odds ratio. These findings are in concordance with other psychooncological studies [10, 13, 14, 17, 18, 19, 20]. The above mentioned variables might be used as indicators for offering and referring to psychooncologic support and a trigger for referrals. Unexpectedly, an advanced stage of the disease had no effect on the care needs. Like other objective medical factors [42], it seemed to have no or little influence on the psychological adjustment of melanoma survivors. The fact that only few of the patients surveyed were in an advanced stage of the disease may have left differences undetected. We had not expected that women have no more need for psychosocial care than men and that neither social class nor education or a steady partnership played a role as this was found in other care need studies, previously [10, 16, 17, 18]. The latter finding may be due to the fact that interactions that can influence care needs are not necessarily caused by the partner but other people around the melanoma patient. Depression and anxiety surprisingly did not determine psychosocial intervention needs. Maybe PHQ und GAD do not capture cancer-related anxiety sufficiently, which might be a reason for being in need for intervention. Besides emotional variables, unfulfilled information needs also were related to the melanoma survivors´ burden. It can be assumed that explanations from the caring physician about illness-related issues are a relatively simple strategy to reduce the burden of the patients. Further studies should direct more attention to this aspect.

### Health care system use and subjective care needs

Only 7% of our melanoma sample used psychological counselling, and only 13% reported any type of psychological support. One fifth reported not using dermatological aftercare, which clearly contradicts the guidelines of the German Cancer Society. Patients with a need for psychosocial intervention had a higher probability of using any psychosocial care, especially psychotherapy or psychological counselling. However, overall rates of utilisation were low, confirming the results of a melanoma study where only 15% of patients with a shorter length of illness received psychosocial support [43]. This is very unsatisfactory given that a substantial proportion of these patients were identified as quite burdened. Maybe melanoma survivors in need of psychosocial intervention do not necessarily seek help themselves and remain undetected. Even survivors at high risk of developing new primary disease did not seek formal emotional support to address melanoma-related concerns [44]. As Winzer et al. [11] summarised, there are many barriers for patients that can explain their avoidance of seeking help: not perceiving a need, not knowing that appropriate help is available, not believing that the intervention helps, not finding help accessible, being embarrassed about seeking help or fearing stigmatisation. Additionally, the health care system is often not sensitive or inquisitive enough to detect the patients´ needs [9]. The need for psychosocial intervention may be underestimated by the medical treatment team as psychosocial issues are not communicated between the survivors and the physicians, the referral pathway is unclear, and funding and resources may not be adequate.

### Limitations

The strengths of this study reside in the fact that the sample was registry-based. All cancer patients are registered in a central cancer registry by law, insuring its representativeness in the state. However, access to patients is complicated by the fact that the registry is not allowed to contact patients directly, requiring cooperation not only from the former patients but also their physicians many years after the cancer diagnosis and treatment. Unfortunately, many missing data regarding initial tumour stage lead to information gaps in the cancer registry. However, given the course of the disease, we did not expect many survivors from progressive disease after 5 years. While response rates are within range of comparable studies (f. e. [14, 45]), the response rate of 52.2% patients in our study poses a limitation. An additional lies in the use of a broad range of standardised self-report scales, including the measurement for ‘need for psychosocial support’. Care utilisation, however, is also self reported, thereby potentially under or overestimating it. Even though this study focused on long-term survivors, no information was available about the patients´ distress levels or their care needs at the time of diagnosis and during the melanoma treatment phase. How, why and when the burdens have developed is not known. This might be of interest, because burdens and care needs may change during the trajectory of melanoma aftercare. Longitudinal (intervention-)studies could enlighten the processes of becoming burdened, the rising and fading of care needs and search of professional help to meet them.

### Relevance for the health care system

Overall, our results show a strong imbalance between unfulfilled care needs and utilisation of the professional cancer care system. Barriers for obtaining adequate care should be identified, and the aftercare should be adjusted to better support long-term melanoma survivors.

The study underscores the need for regular and systematic screening for psychosocial strains and the need for medical aftercare support after melanoma treatment. In order to fulfil psychosocial care needs for melanoma survivors, it is important to consider their care needs from both the health professionals’ perspective as well as the patients’ perspective themselves. Furthermore, it is of interest to identify extensively burdened risk groups by analysing the determinants of being in need for care.

In order to plan health care services for long-term melanoma survivors, attributes of optimal psychosocial care should be defined. Screening with the HQ-S helps to identify distressed patients and may be a substantial first step towards optimising the use of allocated resources. According to the identified needs, it is essential to provide adequate psycho-oncological support including illness related information (see [46]) in order to foster better health-related quality of life and satisfaction with care. Nevertheless, not all unmet needs for support can be fulfilled; for example professional care cannot fill gaps in the social net of the patients. How to effectively direct patients with meetable needs to available services should also receive more attention.
